# Visual disability in neuromyelitis optica spectrum disorders: prognostic prediction models

**DOI:** 10.3389/fimmu.2023.1209323

**Published:** 2023-06-07

**Authors:** Wenqin Luo, Lingyao Kong, Hongxi Chen, Xiaofei Wang, Qin Du, Ziyan Shi, Hongyu Zhou

**Affiliations:** Department of Neurology, West China Hospital, Sichuan University, Chengdu, Sichuan, China

**Keywords:** auqaprin-4, prognosis, disability, prediction model, neuromyelities optica spectrum disorders

## Abstract

**Background and objectives:**

Neuromyelitis optica spectrum disorder (NMOSD) is an autoimmune inflammatory disease of the central nervous system characterized by simultaneous or consecutive episodes of acute optic neuritis and transverse myelitis. Attacks of NMOSD can result in the accrual of severe visual disability over time. This study aimed to develop and validate prognostic models for visual disability risk within 1, 3, and 5 years.

**Methods:**

Medical records of NMOSD patients were retrospectively analyzed. The least absolute shrinkage and selection operator (LASSO) regression algorithm and univariate and multivariate Cox regression analyses were performed to select predictors of visual disability. Two models predicting the probability of visual disability in 1, 3, and 5 years were developed based on different selections and displayed as nomograms. Risk scores were calculated for every patient, and a cut-off point was obtained to recognize patients at high risk.

**Results:**

In total, 161 (25.2%) patients developed visual disabilities during the follow-up period. Four visual disability-related factors were selected using LASSO regression: optic neuritis (ON) onset, higher annual relapse rate (ARR) before maintenance therapy, no maintenance immune suppression therapy (IST), and initial severe attack. Three additional predictors were determined using multivariate Cox regression: male sex, age at first onset, and positive AQP4-IgG serology. Discrimination and calibration were satisfied, with concordance indexes (C-index) close to 0.9 in both models. Decision curve analysis showed good clinical usefulness in both models, and Kaplan-Meier curves showed satisfactory discrimination between patients with high risk and low risk by the cut-off points.

**Conclusion:**

This study reported predictors of visual disability and generated nomograms. High-risk patients need more active treatment and management to avoid unfavorable outcomes.

## 1 Introduction

Neuromyelitis optica spectrum disorder (NMOSD) is an autoimmune inflammatory disease of the central nervous system (CNS) characterized by simultaneous or consecutive attacks of acute optic neuritis (ON) and transverse myelitis (TM) (1). In more than 80% of cases, NMOSD is induced by the release of pathogenic IgG antibodies against auqaprin-4 (AQP4-IgG) (2). Attacks of NMOSD can result in the accrual of severe visual disability over time (1). It has been suggested that within 60 months, unilateral or bilateral blindness occurred in nearly half of patients. Meanwhile, only 25% of disability events were related to the first NMOSD attack (3); thus, recognizing the risk factors for poor prognosis in the early stages is vital for preventing potential disability.

Thus, this study aimed to develop and validate a clinical prognosis prediction model to provide a reference and evidence for clinical practice and patient management.

## 2 Materials and methods

### 2.1 Study design and participants

This study was a retrospective analysis of data from patients prospectively included in the NMOSD cohort at the Medical Center of Southwest China (Department of Neurology, West China Hospital, Sichuan University, China) between January 2015 and January 2022. Patients were included in this study based on the following criteria: (1) NMOSD diagnosis according to the 2015 diagnostic criteria (4) and (2) AQP4-IgG tested using a cell-based assay. Patients that met the following criteria were excluded: (1) incomplete clinical data, (2) follow-up time of less than 6 months, (3) MOG-IgG seropositivity, (4) uncertain treatment records, and (5) refusal to be followed up.

### 2.2 Data collection

All patients diagnosed with NMOSD were registered in our database, and data on the following characteristics were collected: sex, age at onset, presentation of the first attack, concomitant autoimmune disease (including but not limited to systemic lupus erythematosus, inflammatory bowel disease, Sjogren’s syndrome, and rheumatoid arthritis), and maintenance therapy. Face-to-face or video call follow-ups were performed every 3–6 months, and features and presentation of each relapse were recorded. AQP4-IgG serology was detected using a commercial cell-based assay (CBA) (EUROIMMUN AG, Luebeck, Germany) (5, 6) MOG-IgG serology was tested by CBA as previously reported (7). Re-examination of AQP4-IgG was performed after 3–6 months for double-seronegative patients, and the Expanded Disability Status scale (EDSS) was assessed by a trained physician.

Patients who received immunosuppression therapy (IST) were treated with azathioprine (AZA) 2–3 mg/kg/day, mycophenolate mofetil (MMF) 1000–1500 mg/day, or rituximab (RTX) 1000 mg every 6 months, for pediatric patients and elders with weight less than 45 kg, the dose of MMF was adjusted according to the weight (20 mg/kg/day); the dose of RTX was adjusted according to the body surface area (375mg/m^2^), the bods surface area was calculated using Mosteller formula (weight [kg] x height [cm]/3600)½ (8).

### 2.3 Outcome measurement

Disease duration was defined as the interval between the date of first attack and the date of the final follow-up; time before IST was defined as the interval between the date of first attack and the date of initiation of IST, or the disease duration if patients were not treated with immunosuppressor; severe attack was defined as visual score ≥ 5 or EDSS ≥ 6, (the visual score was accessed using Snellen chart at 5 meters, a visual score of 5 was defined as: the worse eye with maximal visual acuity [corrected] less than 20/200 [0.1]) (9, 10); relapse was defined as a new worsening neurological function lasting more than 24 h in the absence of other identifiable causes, or newly found lesions confirmed by MRI, and occurring more than 30 days after a previous attack; active relapse was defined as more than 2 relapses with 12 months (11). Annualized relapse rate (ARR, number of relapses per patient-year) was calculated based on the recorded information; the first attack at onset was not included in the calculation of ARR. Visual disability was defined as a visual score of ≥ 5 points for more than 6 months (9).

### 2.4 Statistical analysis

Categorical variables are presented as frequencies (percentages). Continuous variables are presented as mean ± standard deviation (SD), and if not normally distributed, (Kolmogorov–Smirnov test was applied to verify the normality of distribution of continuous variables), as median (interquartile range, IQR). The mean differences between the two groups were analyzed using unpaired t-tests, while the median differences were evaluated using Mann-Whitney U tests. Categorial variables were compared using Fisher’s exact test.

Penalized likelihood-based methods have drawn much attention recently (12, 13). Because the variables are numerous, to avoid a complex model, predictor selection was performed using the Lasso regression algorithm and 10-fold cross-validation (14, 15). We also performed an exploratory Cox regression analysis, and variables associated with significant changes (p<0.2) in the univariable Cox regression were further analyzed using multivariable Cox regression. Specifically, sex as a demographic variable was included in multivariable Cox regression. A Schoenfeld residual test was performed to evaluate the possible violation of the proportional hazards assumption. To improve the statistical power, treatment with AZA, MMF, and RTX was combined with IST, treatment with oral glucocorticoids (GCs) only, and no therapy was combined with non-IST.

Prediction models were generated according to predictor subsets selected *via* different methods and displayed as nomograms. The C-index was applied to evaluate the discrimination of the models and was visualized *via* time-dependent curves. Calibration curves were created to evaluate the calibration ability of the models, and decision curves analysis (DCA) was performed to evaluate the clinical usefulness. To internally validate the results, we utilized the 0.632 bootstrap method (16) with 1000 resamples and calculated the mean overoptimism and corrected C-index, and also created calibration curves. Finally, the risk scores of all patients were calculated according to different models, and cut-off points were generated to recognize high-risk patients *via* receiver operating characteristic (ROC) analysis. Kaplan–Meier curves and log-rank tests were applied to detect the discrimination ability of cut-off points.

Restricted cubic splines (RCS) with four knots at 5^th^, 35^th^, 65^th^, and 95^th^ centiles were used to flexibly model the association between onset age and the risk of visual disability, analysis of variance (ANOVA) was used to test the statistical significance of non-linear correlation.

All statistical analyses were performed using R software (version 4.0.3; R Foundation for Statistical Computing, Vienna, Austria; http://www.r-project.org/) with rms, ggDCA, survivalROC, glmnet, and survival packages. Statistical significance was set at p-values of < 0.05.

### 2.5 Standard protocol approvals and patients’ consent

This study was approved by the Ethics Committee of Sichuan University (approval number: 2018 SHEN 29). Written informed consent was obtained from all patients.

## 3 Results

### 3.1 Study population

**Figure 1:**
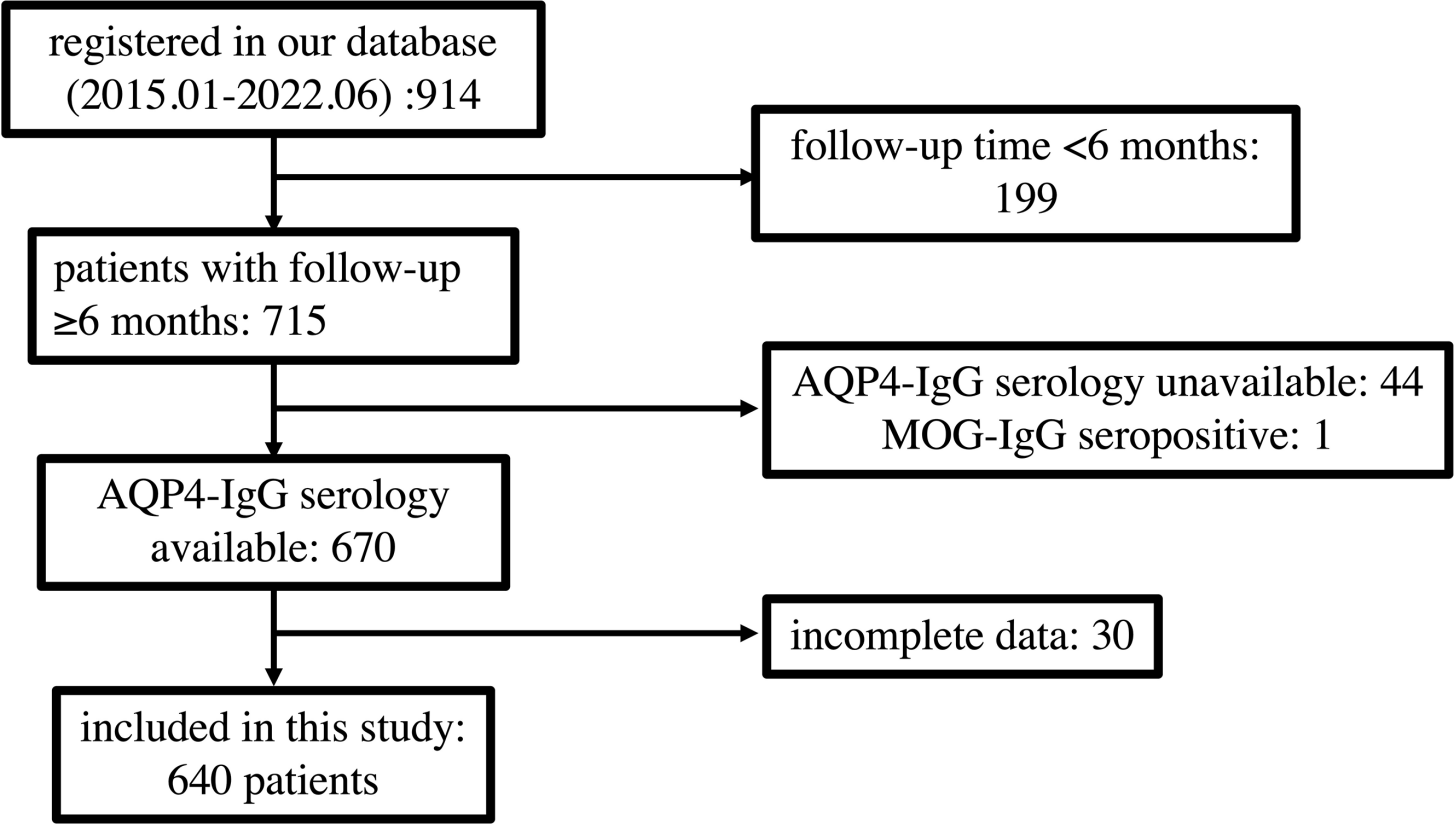
Flow chart depicting participant eligibility. AQP4, aquaporin 4; MOG, myelin oligodendrocyte glycoprotein.

Of 914 patients registered in our database between January 2015 and June 2022, 274 were excluded, and 640 patients were ultimately included in this study. The inclusion and exclusion flows of participants are shown in **Figure 1**.

### 3.2 Demographic and clinical characteristics of participants

**Table 1** summarizes the demographic and clinical characteristics of the study population. The cohort was characterized by several demographic features. The mean onset age was 38 ± 14 years (range 8 to 76 years), and 87% of the population was female. The majority of the cohort were Han Chinese, while 24 (3.8%) patients were Tibetans. The median follow-up time was 68 months. Seventy-one (11%) patients had concomitant autoimmune diseases, and 572 (89%) patients were AQP4-IgG seropositive. A total of 452 (70.6%) patients received maintenance IST during follow-up, and 188 (29.4%) patients did not receive maintenance therapy.

**Table 1:** Demographic and clinical characteristics of the cohort.

Characteristic	Total N = 640	Visual-disability-free N = 479	Visual disability N = 161	p-value
**Sex**				0.58
Female	559 (87%)	416 (87%)	143 (89%)	
Male	81 (13%)	63 (13%)	18 (11%)	
**Ethnicity**				0.16
Han Chinese	616 (96%)	464 (97%)	152 (94%)	
Tibetan	24 (3.8%)	15 (3.1%)	9 (5.6%)	
**Age at first onset** **(range 8-76 years)**	38 ± 14	38 ± 14	39 ± 15	0.34
**Age group**				0.074
<20	68 (11%)	46 (9.6%)	22 (14%)	
<30	122 (19%)	93 (19%)	29 (18%)	
<40	140 (22%)	116 (24%)	24 (15%)	
<50	167 (26%)	123 (26%)	44 (27%)	
>=50	143 (22%)	101 (21%)	42 (26%)	
Comorbidity				
Hypertension	32 (5.0%)	26 (5.4%)	6 (3.7%)	0.53
Autoimmune disease	71 (11%)	55 (11%)	16 (9.9%)	0.66
Diabetes	21 (3.3%)	17 (3.5%)	4 (2.5%)	0.62
**ON onset**	255 (40%)	139 (29%)	116 (72%)	<0.001
**AQP4 IgG serology**				0.017
Negative	68 (11%)	59 (12%)	9 (5.6%)	
Positive	572 (89%)	420 (88%)	152 (94%)	
**Maintenance therapy**				<0.001
No IST	188 (29.4%)	69 (14%)	119 (74%)	
IST	452 (70.6%)	410 (86%)	42 (26%)	
Initial severe attack	187 (29%)	107 (22%)	80 (50%)	<0.001
Active relapse before IST	334 (52%)	240 (50%)	94 (58%)	0.083
ARR before IST	0.48 (0.09, 1.00)	0.44 (0.00, 1.00)	0.59 (0.24, 1.00)	0.006
Disease duration (months)	68 (36, 119)	63 (34, 114)	84 (51, 139)	<0.001

Data are presented as n (%), mean ± standard deviation, or median (interquartile range). AQP4-IgG, aquaporin-4 IgG antibody; ON, optic neuritis; TM, transverse myelitis; ARR, annual relapse rate; IST, immunosuppressive therapy.

One hundred and sixty-one (25.2%) patients developed visual disabilities. The differences in sex, onset age, ethnicity, and comorbidity were not statistically significant between disabled and non-disabled patients. Compared to those who did not develop visual disability, the disabled patients had a higher ratio of optic neuritis as the presentation of onset (p <0.001) and a higher proportion of AQP4-IgG seropositive (p=0.017). Most (86%) of patients with non-visual disabilities received IST, while the percentage was significantly lower (26%) in patients with visual disabilities (p <0.001). Patients with visual disability showed a higher ratio of suffering a severe attack at the first onset and experiencing more frequent relapses before IST initiation.

We additionally analyzed visual outcomes in different age groups, the results are seen in **Supplementary Table 1**. The highest proportion of patients who developed visual disability was observed in patients aged < 20 years, and that proportion decreased in the following 2 age groups, and then increased in the last 2 age groups, although the differences among groups were not statistically significant. RCS (**Supplementary Figure 1**) suggested a non-linear correlation between onset age and risk of visual disability, the S-shaped curve suggested a decrease in risk of visual disability over age, and the risk increased after onset age > 30 years, and finally stay flat in patients over 55 years (p for non-linear association = 0.024).

### 3.3 Predictor selection and model development

Fourteen candidate variables were included in the selection, and the event per variable (EPV) was 11.5 (14/161), suggesting that the estimation of average risk was reliable (17, 18).

**Figure 2:**
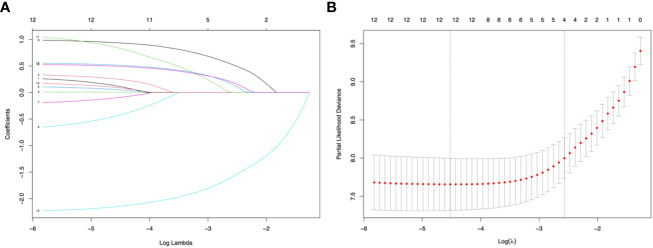
Lasso regression and 10-fold cross-validation for predictor selection. **(A)** The path of the parameter estimated over a grid of values for λ. **(B)** 10-fold cross-validation. Minimum cross-validation error reached when the log of λ is -4.52 (corresponding the left line); when log of λ = -2.57, the error is the minimum error plus 1 standard error (corresponding the right line), and the number of selected variables is 4.

Four predictors were selected using Lasso regression: ON onset (Lasso’ *β*=0.52), ARR before therapy (Lasso’ *β* =0.15), IST (Lasso’ *β*=-1.58), and initial severe attack (Lasso’ *β*=0.19) (**Figures 2A, B**).

We performed an exploratory univariate Cox regression and a subsequent multivariate Cox regression. The effects of the predictors are summarized in **Table 2**. Patients with older age at onset had a higher risk of developing visual disability (hazard ratio [HR]=1, 95% confidence interval [CI] 1-1.02, p=0.045); ON onset was an extremely strong predictor for visual disability (HR=2.91, 95% CI 1.99-4.24, p<0.001); compared to AQP4-IgG seronegative patients, the seropositive patients had significantly higher risk of visual disability (HR=2.98, 95% CI 1.48-5.98, p=0.002); maintenance IST decreased the risk of visual disability by 91% (HR=0.09, 95% CI 0.07-0.15, p<0.001); however, clinical features before IST initiation still predict the visual outcome: initial severe attack and higher ARR before IST were both identified as risk factors for visual disability.

**Table 2:** Univariable and multivariable cox regression for visual disability.

	univariable analysis			multivariable analysis		
Characteristics	HR	95% CI	p	HR	95% CI	p
sex						
Female (Ref.)	1	–	–	–	–	–
Male	1.05	0.64-1.72	0.845	1.36	0.82-2.26	0.235
Race						
Han Chinese (Ref.)	1	–	–	1	–	–
Tibetan	1.97	1-3.86	0.049	1.66	0.83-3.32	0.153
**Age at first onset**	1.02	1-1.03	0.007	1.01	1-1.02	0.045
Comorbidity						
Hypertension	0.71	0.31-1.61	0.412	–	–	–
concomitant autoimmune disease	0.86	0.51-1.44	0.567	–	–	–
diabetes	0.63	0.23-1.69	0.356	–	–	–
**ON onset**	4.57	3.24-6.45	<0.001	2.91	1.99-4.24	<0.001
AQP4-IgG serology						
Double negative (Ref.)	1	–	–	1	–	–
AQP4 IgG seropositive	2.3	1.18-4.52	0.015	2.98	1.48-5.98	0.002
Maintenance therapy						
No IST (Ref.)	1	–	–	1	–	–
IST	0.09	0.07-0.14	<0.001	0.1	0.07-0.15	<0.001
**ARR before IST**	1.51	1.24-1.85	<0.001	1.85	1.54-2.23	<0.001
**initial severe attack**	3.33	2.44-4.54	<0.001	1.93	1.38-2.69	<0.001
**active relapse before IST**	1.21	0.88-1.66	0.231	–	–	–

HR, hazard ratio; ON, optic neuritis; TM, transverse myelitis; ARR, annual relapse rate; AQP4, aquaporin 4; IST, immunosuppressive therapy.

**Figure 3:**
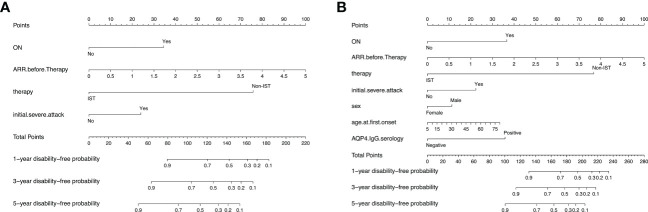
Nomograms for predicting 1-, 3-, and 5-year visual-disability-free of 2 models in the development cohort. **(A)** nomogram for model 1 (based on Lasso regression). The apparent C-index is 0.88 (95% CI: 0.85-0.91). **(B)** nomogram foe model 2 (based on multivariable cox regression). The apparent C-index is 0.89 (0.86-0.92). ON, optic neuritis; ARR, annual relapse rate; AQP4, aquaporin 4.

Subsequently, two prediction models were generated and presented as nomograms (model 1, based on variables selected by Lasso; and model 2, based on variables selected by multivariable Cox regression). The nomograms for 1-, 3-, and 5-year visual-disability-free probability were depicted in **Figure 3**. Lasso identified ON onset, higher ARR before IST initiation, initial severe attack, and no IST as predictors of visual disability. Three additional predictors were identified by multivariable Cox regression: male sex, older age at onset, and seropositive AQP4-IgG.

### 3.4 Internal validation of prediction models for visual disability

**Figure 4:**
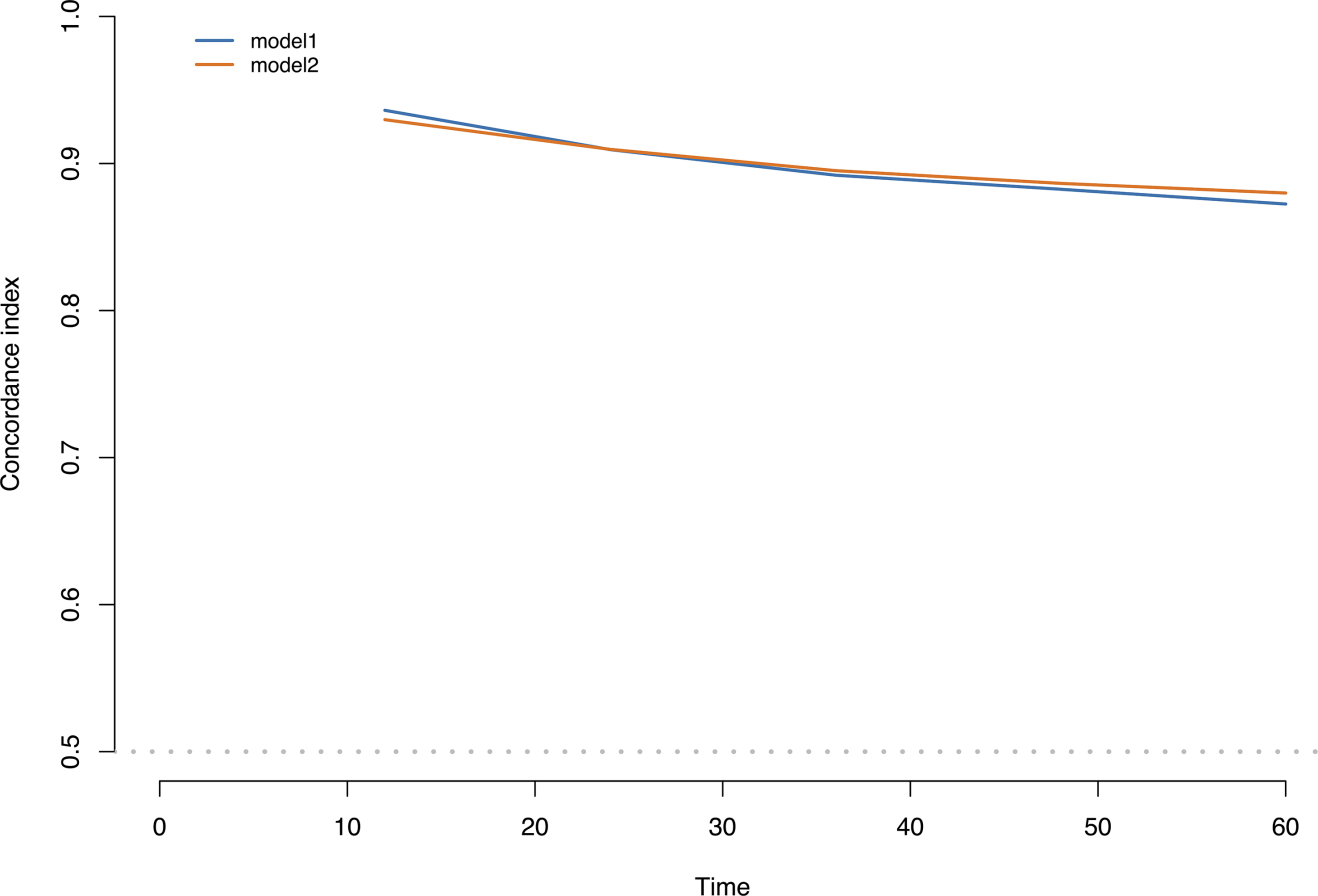
Time-dependent C-index curves of 2 models. Model 1 had a higher discrimination in the first 2 years while C-index of model 2 was higher in the last 3 years. C-index of both models decreased over time.

**Figure 5:**
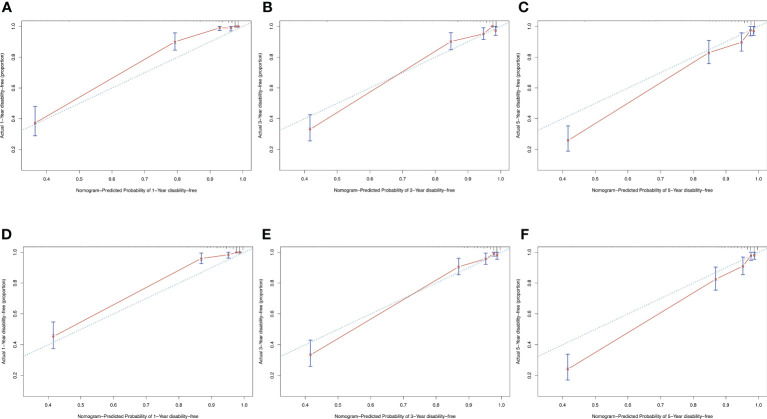
Calibration curves of both models showed a satisfied agreement between predicted and actual probability in 1-, 3-, and 5-years. **(A–C)** calibration curves of model 1 for predicting 1-, 3- and 5-year-visual-disability-free probability. **(D–F)** calibration curves of model 2 for predicting 1-, 3- and 5-year-visual-disability-free probability.

**Figure 6:**
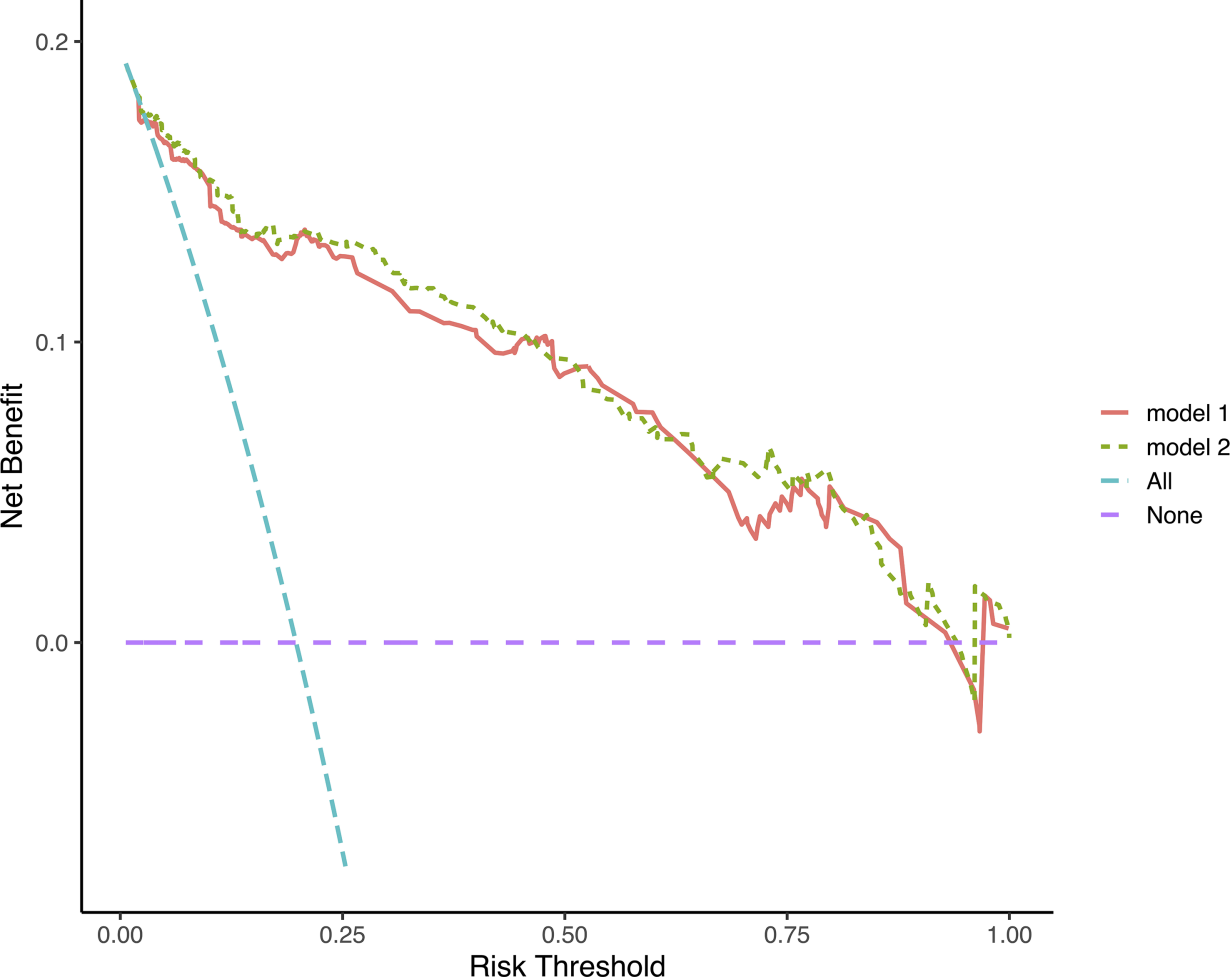
Decision curves analysis of 2 models. The decision curves indicated that net benefit was assured in a wide range of risk threshold, both nomograms was superior to treat-all or treat-none strategy.

Discrimination ability was evaluated using 0.632 bootstraps with 1000 resamplings. Model 1 showed an overoptimism of 0.0034 and a corrected C-index of 0.88. In contrast, model 2 showed an overoptimism of 0.0074 and a corrected C-index of 0.88. The time-dependent C-index curves (**Figure 4**) showed a decrease in the C-index over time but the model remained robust with C-index of 0.85. The C-index of Model 1 was relatively higher in the previous two years and reversed in the last three years. Calibration curves demonstrated good agreement between the predicted and actual 1-, 3- and 5-year visual-disability-free probabilities in both models (**Figures 5A–F**). DCA demonstrated that if the threshold probability of patients is between 0.1 to 0.9, both models presented superior net benefits for predicting visual disability compared to treat-all or treat-non strategy (**Figure 6**).

**Figure 7:**
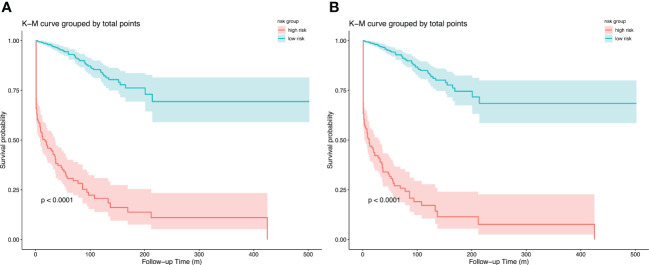
Kapan-Meier curves of visual disability in primary cohort for **(A)** model 1, cut point is 89.5, and **(B)** model 2, cut point is 147.6; stratified by risk group.

In both models, the risk scores of each patient were calculated, and an optimal cut-off point for distinguishing between patients with low and high risk in 1 year was generated using ROC analysis. Patients were divided into high-risk and low-risk groups according to their scores. Kaplan-Merier curves (**Figure 7**) showed good discrimination between those with high and low risk of visual disability. In both models, the cutoff point was 89.5 (the corresponding 1-year visual disability probability according to the nomogram is 0.15, and the corresponding net benefit according to DCA is 0.138) and 147.6 (the corresponding 1-year visual-disability probability according to nomogram is 0.15, and the corresponding net benefit according to DCA is 0.136), respectively.

## 4 Discussion

Several studies have revealed the risk factors for permanent visual disability in NMOSD (19–22), while the effect of some features on the visual outcome remained debated. In a British cohort, younger age was linked to a higher risk of visual disability (21), while a Chinese cohort identified older age as a risk factor for visual disability (19). Meanwhile, a prediction model is necessary for individual management in clinical practice. Thus, we aimed to explore the valuable predictors of visual disability and to develop a clinical prediction model. The current study systematically explored the potential predictors of visual disability and developed two prediction models using different selection strategies in a large cohort of patients with NMOSD (including AQP4-IgG seropositivity and AQP4/MOG-IgG double-negative patients). We identified age of onset, AQP4-IgG serology, IST status, ON onset, ARR before IST initiation, and initial severe attack as predictors of visual disability.

Within our cohort, 70.6% of patients received IST. Compared to the disability-free patients, patients who developed visual disability had a lower ratio to receive IST, the results of regression also confirmed that IST decreased the risk of visual disability. Nevertheless, there was frequently a lapse between the initial attack or NMOSD diagnosis and the initiation of IST, and during this interval, patients could experience multiple relapses and develop visual disability. These observations underscore the importance of prompt and accurate diagnosis, as well as the timely initiation of maintenance IST in the early stages of NMOSD onset, as a means of reducing the risk of disability. In addition, some patients developed visual disabilities during their first attack of NMOSD, which is clearly beyond the scope of any intervention. Early initiation of IST helps to prevent the progression to bilateral blindness in patients who have experienced unilateral blindness, particularly those with severe ON attacks.

Given the rarity of MOG-IgG-seropositive patients in our cohort, these patients were excluded, and AQP4-IgG seronegative patients were included. In the current cohort, AQP4-IgG was found in 89% of the patients, which is consistent with a previous study (2). Although some studies suggested that EDSS at the last follow-up in double-seronegative patients was not different from that in AQP4-IgG seropositive patients, only a few studies described the visual outcome of AQP4-IgG seropositive and double negative patients. Our previous study has revealed a higher risk for visual disability in AQP4-IgG seropositive patients compared to double-seronegative patients (23). In the present study, AQP4-IgG serology status remains a strong predictor for visual disability, indicating that the test of serology status at the early stage of onset is necessary. Considering the possibility of a false-negative AQP4-IgG test and the poor outcomes of AQP4-IgG seropositive patients, reexamination of serology status must be considered for double-seronegative patients.

A previous cohort study suggested that early age of onset (<21 years) was related to a worse visual outcome (24). In our cohort, the proportion of patients who developed visual disability was higher in patients with onset age less than 20 years, consistent with the previous study, although multiple comparisons were not performed. However, onset age was a predictor of visual disability in our models; every additional 1 year of onset age increased the risk of visual disability by 1%, suggesting a potential nonlinear correlation between onset age and visual disability, the results of RCS (**Supplementary Figure 1**) confirmed the hypothesis. Given to the relatively small size of patients aged < 20 years (N = 68), future studies are needed to furtherly explore the correlation between onset age and visual outcome. In addition, it should be noted that some studies reported that the age of onset tends to be younger in patients presenting with ON (21, 25),and in our cohort, ON onset was a major risk factor. This finding partially explained the higher risk of visual disability observed in the younger population. ON and TM were the most common presentations of NMOSD attacks, although there was no evidence that the mixed presentation of ON and TM was related to a higher risk of visual disability in the present study.

Quan et al. have reported that the EDSS at onset is a predictor of visual outcomes (24). In the current study, severe attacks were defined using a stricter definition yet still showed an important predictive value. Severe attack was only defined according to the visual accuracy and EDSS, and no distinction was made between severe attacks of ON or TM. It is unclear whether severe attacks of TM at onset are related to a poor visual outcome. Further studies are required to confirm this correlation.

Based on two selection strategies, we generated two prediction models and nomograms. After 0.632 bootstrap with 1000 resampling and adjusting the overoptimism, the corrected C-index demonstrated a satisfying discrimination ability (0.88 in both nomograms), and time-dependent curve of C-index indicated a slightly better discrimination of model 1 in the first 2 years, while the discrimination of model 2 was superior in the last 3 years, suggesting that model 2 is better suited for predicting the long-term outcome. Calibration curves exhibited good agreement between predicted and actual probability of visual disability. The best agreement was reached in predicting 3-year visual-disability-free probability. By applying the DCA method, we found that both nomograms demonstrated clinical utility and outperformed the treat-all (or in NMOSD, treat-all with potent IST) or treat-none (or treat with low efficacy therapy) strategies. We also provided an optimal cut-off point to recognize patients at high risk of visual disability, and the KM curves clearly separated patients with high and low risks of visual disability.

Our study had several limitations. A major limitation is the lack of external validation, limiting the extrapolation of our models. Another limitation was that pediatric patients were not excluded in our cohort, which would have extended the population application of our models. The disease course of pediatric patients and adult patients differ, which can lead to reduced predictive accuracy when attempting to model the outcomes of these groups separately. The final limitation was the retrospective design; which resulted inevitable bias, future prospective studies are needed to confirm our findings.

In conclusion, the present study analyzed the predictors of visual disability; AQP4-IgG seropositivity, ON as presentation at first attack, and older onset age as strong risk factors for visual disability. For these patients, high-effect immunosuppression therapy needs to be performed at an early stage.

## Data availability statement

The raw data supporting the conclusions of this article will be made available by the authors, without undue reservation.

## Ethics statement

The studies involving human participants were reviewed and approved by Ethics Committee of Sichuan University. Written informed consent to participate in this study was provided by the participants’ legal guardian/next of kin.

## Author contributions

WL: study design, statistical analysis, and manuscript writing; ZS and XW: study design and data collection; HC, LK, and QD, data collection; HZ: study design and statistical analysis. All authors contributed to the article and approved the submitted version.
