# Supplementary material for: Visual disability in neuromyelitis optica spectrum disorders: prognostic prediction models
Source: Front Immunol. 2023 Jun 7;14:1209323. doi: 10.3389/fimmu.2023.1209323 (PMC10282746; doi:10.3389/fimmu.2023.1209323)
Supplement: Supplementary file 1 [file DataSheet_1.docx]

**Supplementary Table 1. Visual outcome in different age groups**

| **Characteristic** | **<20, N = 68** | **<30, N = 122** | **<40, N = 140** | **<50, N = 167** | **>=50, N = 143** | **p-value** |
| --- | --- | --- | --- | --- | --- | --- |
| **Sex** |  |  |  |  |  | 0.67 |
| female | 63 (93%) | 106 (87%) | 121 (86%) | 143 (86%) | 126 (88%) |  |
| male | 5 (7.4%) | 16 (13%) | 19 (14%) | 24 (14%) | 17 (12%) |  |
| **ON onset** | 31 (46%) | 62 (51%) | 49 (35%) | 63 (38%) | 50 (35%) | 0.039 |
| **AQP4 IgG serology** | |  |  |  |  | 0.002 |
| Negative | 4 (5.9%) | 14 (11%) | 28 (20%) | 11 (6.6%) | 11 (7.7%) |  |
| Positive | 64 (94%) | 108 (89%) | 112 (80%) | 156 (93%) | 132 (92%) |  |
| **Visual disability** | 22 (32%) | 29 (24%) | 24 (17%) | 44 (26%) | 42 (29%) | 0.074 |

AQP4, aquaporin 4; ON, optic neuritis.

**Supplementary Figure 1. Non-linear correlation between onset age and risk of visual disability.**


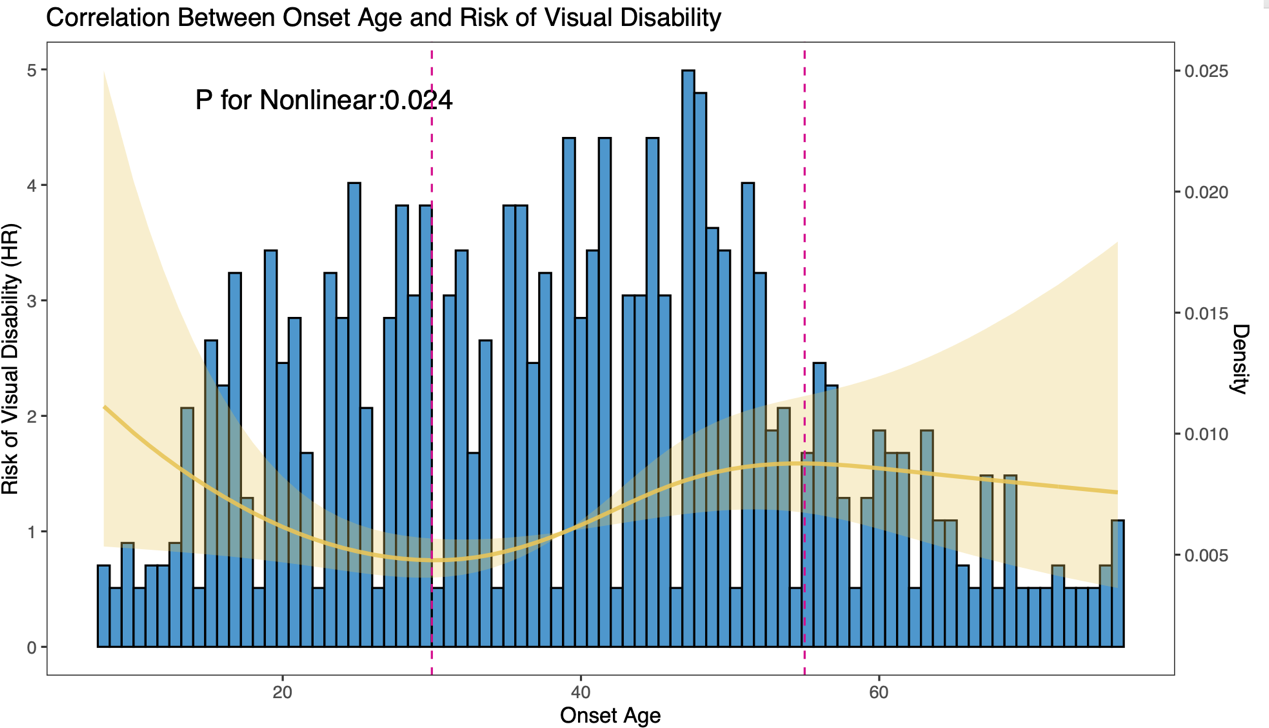


Adjusted for sex, AQP4-IgG serology status, ON onset, therapy status, ARR before therapy, and initial severe attack. Hazard ratios are indicated by solid lines and 95% CIs by shaded areas. vertical dotted lines indicate inflexion points of the curve (aged 30 years and 55 years). Blue histogram indicates the frequency in each age. AQP4, aquaporin 4; ARR, annualized relapse rate; ON, optic neuritis.
